# A Cost-Effectiveness Analysis of a Ketamine-assisted Psychotherapy Program Compared to Online Group Psychotherapy in British Columbia, Canada

**DOI:** 10.1192/j.eurpsy.2024.652

**Published:** 2024-08-27

**Authors:** V. Tsang

**Affiliations:** UBC, Vancouver, Canada

## Abstract

**Introduction:**

Depression continues to present significant economic burdens to the Canadian healthcare system. Novel therapies, including those that incorporate psychoactive substances such as ketamine, present an opportunity to evaluate both clinical and economic effectiveness against current standards of care, which may be repeatedly proving ineffective in treating depression for some individuals.

**Objectives:**

This paper evaluates the cost-effectiveness of the Roots to Thrive ketamine program compared to group psychotherapy covered through the medical services plan in British Columbia, Canada.

**Methods:**

A discrete-time Markov-model is used to estimate depressive states over five cycles for a treatment cohort and a synthetic control cohort. The transition probabilities for the treatment cohort are calculated from Roots to Thrive program data (n = 62) over the past 3 years, with the control cohort using published values from the literature. Both cohorts use the same starting state distribution, excess healthcare utilization rates for each severity level of depression, and utility outcomes based on depression state severity.

**Results:**

Compared to the control cohort, the Roots to Thrive program was less expensive and produced better outcomes as measured by PHQ-9 scores and Quality-Adjusted life years over 5 treatment cycles. On average, the Roots to Thrive program would save $14,481 and produce 0.94 additional QALY’s per individual compared to group psychotherapy of three patients per provider in the current standard of care.

**Conclusions:**

From an economic perspective, incorporating the Roots to Thrive program - or a program like it - into care in British Columbia would provide both an improvement in health outcomes and reduce expenditure by the ministry of health. These funds could be reinvested into other areas of the healthcare system to improve the lives of all British Columbians, even those that do not engage in psychedelic-assisted psychotherapy.

**Disclosure of Interest:**

None Declared

